# Transmigration of Neutrophils From Patients With Familial Mediterranean Fever Causes Increased Cell Activation

**DOI:** 10.3389/fimmu.2021.672728

**Published:** 2021-05-17

**Authors:** Anush Martirosyan, David Poghosyan, Susanna Ghonyan, Nune Mkrtchyan, Gayane Amaryan, Gayane Manukyan

**Affiliations:** ^1^ Laboratory of Molecular and Cellular Immunology, Institute of Molecular Biology National Academy of Sciences of the Republic of Armenia (NAS RA), Yerevan, Armenia; ^2^ National Pediatrics Center of Familial Mediterranean Fever “Arabkir” Joint Medical Center- Institute of Child and Adolescent Health, Yerevan, Armenia; ^3^ Department of Pediatrics, Yerevan State Medical University, Yerevan, Armenia

**Keywords:** Familial Mediterranean fever, neutrophils, fresh/aged neutrophils, transmigration, IL-1β, immunophenotype, FcγRs

## Abstract

Familial Mediterranean fever (FMF) is caused by pyrin-encoding *MEFV* gene mutations and characterized by the self-limiting periods of intense inflammation, which are mainly mediated by a massive influx of polymorphonuclear neutrophils (PMNs) into the inflamed sites. Perturbation of actin polymerization by different pathogens was shown to activate the pyrin inflammasome. Our aim was to test whether cytoskeletal dynamics in the absence of pathogens may cause abnormal activation of PMNs from FMF patients. We also aimed to characterize immunophenotypes of circulating neutrophils and their functional activity. Circulating PMNs displayed heterogeneity in terms of cell size, granularity and immunophenotypes. Particularly, PMNs from the patients in acute flares (FMF-A) exhibited a characteristic of aged/activated cells (small cell size and granularity, up-regulated CXCR4), while PMNs form the patients in remission period (FMF-R) displayed mixed fresh/aged cell characteristics (normal cell size and granularity, up-regulated CD11b, CD49d, CXCR4, and CD62L). The findings may suggest that sterile tissue-infiltrated PMNs undergo reverse migration back to bone marrow and may explain why these PMNs do not cause immune-mediated tissue damage. A multidirectional expression of FcγRs on neutrophils during acute flares was also noteworthy: up-regulation of FcγRI and down-regulation of FcγRII/FcγRIII. We also observed spontaneous and fMPL-induced activation of PMNs from the patients after transmigration through inserts as seen by the increased expression of CD11b and intracellular expression of IL-1β. Our study suggests heightened sensitivity of mutated pyrin inflammasome towards cytoskeletal modifications in the absence of pathogens.

## Introduction

Familial Mediterranean fever [FMF, MIM249100] is an autoinflammatory syndrome characterized by the recurrent episodes of fever and aseptic polyserositis. Gain-of-function mutations in the MEFV gene which encodes pyrin are the cause for FMF (1, 2). Interaction of the N-terminal part of pyrin with microtubules and co-localization of pyrin with actin (3, 4) suggest that pyrin might be a sensor for actin homeostasis (5). Later on, pyrin was shown to recognize downstream Rho modifications, most likely involving the actin cytoskeleton modifications (6, 7). Pyrin has been shown to activate caspase-1 and mature IL-1β and IL-18 release in response to Rho-inactivating toxins from a number of pathogenic bacteria (5, 6, 8). The involvement of cytoskeleton might be confirmed by the therapeutic efficiency of colchicine, antimitotic drug, which causes microtubule disruption/depolymerization as well as re-organization of actin cytoskeleton (9, 10).

The pyrin is primarily expressed in neutrophils (polymorphonuclear cells, PMNs), eosinophils, and cytokine-activated monocytes (11). Despite the main players of self-limited inflammatory attacks are neutrophils, remarkably little is known about their phenotypic and functional characteristics. PMNs carrying *MEFV* mutations display characteristics of activation status (12, 13). Increased spontaneous release of IL-18, S100A12, myeloperoxidase (MPO), caspase-1, and proteinase 3 have been shown by neutrophils from FMF patients. Moreover, the highest levels of IL-18 were released by the cells derived from the patients with homozygous M694V mutations (14). In our previous experiments, we have observed a phenomenon of heightened sensitivity of neutrophils from FMF patients towards *in vitro* conditions in the inductor-free media (13, 15). Similar results with the use of monocytes have been obtained later by another group (16). *In vitro* experiments unavoidably impose mechanical forces on cells that may affect cytoskeletal structure and modulate cellular behaviour (17). It is already known that mechanical deformation has the ability to increase expression of adhesion molecules, reorganize cell cytoskeleton, increase free intracellular Ca2+ concentration, and induce cell activation (18, 19). The routine experimental procedures involving external mechanical forces applied to the cells may lead to the generation of danger signals that are sensed by the pyrin inflammasome. Recent investigations showing the pyrin inflammasome activation in response to actin modifications (6, 7), together with our results suggesting the excessive activation of neutrophils from FMF patients in *ex vivo* experiments (13, 15), warrants further studies of pyrin-cytoskeleton interactions. We hypothesize that mechanical forces induced by the transmigration of the cells may enhance pyrin inflammasome priming. To address our hypothesis and evaluate the role of cytoskeletal dynamics in increased activation of the cells, we performed a series of *in vitro* assays where PMNs were influenced by different stressful signals such as transmigration through Transwell insert, stress hormone epinephrine, and bacterial ligands. In parallel, immunophenotypes of circulating neutrophils and their functional activity were analysed.

## Materials and Methods

### Patients

A total of 35 patients with fulfilled Tel Hashomer criteria for FMF diagnosis and at least one mutation in the *MEFV* gene were enrolled in the study (20). All FMF patients were recruited at the”Arabkir” Medical Center – Institute of child and adolescent health (Arabkir MC-ICAH, Yerevan, Armenia). Fourteen of the 35 patients expressed a typical FMF attack and were identified as FMF-A group, and the remaining 21 clinically asymptomatic FMF patients in attack-free period were identified as FMF-R group. All patients within FMF-R groups were receiving colchicine, while the patients from FMF-A group were newly diagnosed colchicine-naive patients. Determination of the acute phase was based on clinical and laboratory findings (fever, FMF-related symptoms such as abdominal and/or thoracic pain, arthritis, C-reactive protein, white blood cell counts, erythrocyte sedimentation rate, etc.). The remission phase was defined as being free of attacks for at least 3 months. The control group consisted of 20 healthy, age and gender-matched volunteers without any concomitant chronic disorders. The potential carriage of *MEFV* mutations within the control group was not assessed, however, healthy individuals with family history of FMF in three successive generations were excluded from the study. The clinical and demographic characteristics of the participants involved in the study are summarized in **Table 1**. The study was approved by the Ethical Committee of the Institute of Molecular Biology NAS RA (IRB IORG0003427). Written informed consent was obtained from all adult participants or from parents/legal guardians of participants under 18.

**Table 1:** Demographic and clinical characteristics of FMF patients and healthy donors.

	HD	FMF-R	FMF-A
***№ of participants***	20	21	14
***Age***	21 (16–20)	16 (10–18)	15 (7–18)
***Leucocytes*** [10*9/L]	7.36 ± 1.95	7.57 ± 1.91	9.51 ± 2.61
***PMNs*** [10*9/L]	3.95 ± 2.18	4.73 ± 1.54	6.58 ± 2.88
***PMNs*** [%]	49.2 ± 6.48	56.4 ± 7.36	65.15 ± 11.97
***CRP*** [mg/dL]	0.9 ± 1.31	3.26 ± 4.65	101.08 ± 89.16
***ESR*** [mm/hr]	7.3 ± 4.62	9.71 ± 5.67	15.42 ± 11.02
***Colchicine treatment***	0/20	21/21	0/14
***MEFV mutations***	no family history of FMF	2 homozygous/ 19 heterozygous	4 homozygous/ 10 heterozygous

### Cell Surface Staining

Peripheral venous blood samples were drawn from patients with FMF and healthy donors (HD) into EDTA- and Heparin-containing tubes and processed within 2h. To investigate the expression of cell surface markers in PMNs, whole blood was aliquoted (50 μl per tube) and stained with optimal concentrations of fluorochrome-conjugated monoclonal antibody combinations directed against the following antigens: CD11b, CD15, CD16, CD32, CD49d, CD62L, CD64, and CD184 (CXCR4) for 20 minutes at room temperature, in the dark. Isotype matched FITC, PE, PE/Cy7, APC-conjugated irrelevant antibodies were used to establish background staining. PMNs population was identified through sequential gating strategy: based on forward scatter (FSC), side scatter (SSC), and CD15 expression. Data was acquired on a FACSCalibur flow cytometer (BD) equipped with BD CellQuest™ Pro acquisition software and analysed using the FlowJo vX0.7 software (Tree Star, Inc, San Carlos, CA). At least 10,000 total events were collected per sample. Cell viability was verified with PI staining. Results are expressed as the percentage and median fluorescence intensity (MFI) of the cells for each examined marker.

### Oxidative Burst Assay

To determine PMNs oxidative burst capacity, dihydrorhodamine 123 (DHR-123) (Sigma-Aldrich) conversion into the fluorophore rhodamine was evaluated. Briefly, the aliquots of heparinized whole blood were incubated for 20 min with DHR-123 at final concentration 10 μM in the dark at 37°C. fMLP (100 ng/ml) and as a positive control PMA (50 ng/ml) were added to the corresponding tubes and incubated for further 30 min in the dark at 37°C. As negative control cells were left in the RPMI medium alone. Reaction was stopped by placing tubes on ice for 10 min. Following erythrocytes lysis with cold hypotonic solution and a single washing step, fluorescence intensity of the cells was immediately analysed by flow cytometry.

### Phagocytosis

PMNs phagocytosis capacity was assessed in aliquots (50 μl per tube) of heparinized whole blood, stimulated with following inducers: LPS (100 ng/ml), PGN (1 μg/ml) in the dark at 37°C for 60 min. Afterward, cells were incubated with 0,5 μl (density: 4.55x10^10^ particles/ml) microbeads (Polysciences, Inc., Fluoresbrite^®^ YG Microspheres 1.00 µm size) for 30 min at 37°C. For negative control, cells were incubated with microbeads at 4°C for 30 min, without prior stimulation. Following the incubation, cells were washed with ice-cold PBS to remove any free (non-ingested) particles and analysed by flow cytometry.

### Neutrophil Transmigration Assay

To analyse whether mechanical stress abnormally affects cell activation, we performed a transmigration assay. Briefly, PMNs were isolated with Histopaque-1077 gradient (Sigma, St. Louis, MO) and subjected to transmigration using 24-well plate and 6.5-mm Transwell Inserts with 3-μm pore Polyester Membrane (Corning, NY, USA). Inserts (upper compartment) were placed into the wells containing 600 μl RPMI-1640 supplemented with 0.5% bovine serum albumin (BSA) in the absence or presence of fMLP (100 ng/ml). PMNs were suspended at final concentration 1x10^6^/100 μl, loaded into the upper compartment and allowed to transmigrate for 2h, at 37°C under a humidified atmosphere of 5% CO_2_. In parallel, PMNs were cultured at the same conditions in a 24-well plate without inserts as a non-migrated control. Following cultivation period, migrated and non-migrated cells were surface-labelled with CD11b, fixed/permeabilized with Fixation Buffer/Permeabilization Wash buffer (BioLegend), blocked to avoid non-specific binding, stained intracellularly with IL-1β for 20 min, and analysed with flow cytometry. Supernatants of migrated and non-migrated cells were collected and stored at -80°C for further IL-1β quantification.

### Actin Polymerization Assay

Isolated PMNs (500.000 cells per measurement) were stimulated with fMLP (100 ng/ml) or left untreated for 5, 15, 30, 60, 120 and 180 sec. For every time point, regardless of stimulation, reaction was stopped by adding a Fixation Buffer (BioLegend) for 20 min at room temperature. Thereafter, cells were permeabilized and blocked to avoid non-specific binding. Following 20 min incubation, cells were intracellularly stained with FITC-conjugated Phalloidin (Abcam, 1:1000). As negative control, PMNs were left either unstimulated or unstained. Following 30 min incubation in the dark at 4°C, actin polymerization was determined by the level of Phalloidin expression, as measured by flow cytometry.

### Kinetic of Cultured PMNs

Time-dependent dynamics of neutrophils activation in the absence of any external forces were analysed using isolated PMNs (1x10^6^) cultured in a complete RPMI medium separately for 1h, 2h, 3h and 4h. After each hour, cell culture supernatants were aspirated and stored for further IL-1β analysis. The cells were then surface-labeled with CD11b, fixed/permeabilized and stained intracellularly with IL-1β for 20 min. Samples were acquired on the flow cytometer.

### Whole Blood Cultivation With Epinephrine

Aliquots of 50 μl whole blood were cultured in the absence or presence of Epi (10, 100, 1000 μM), LPS (100 ng/ml) or LPS (100 ng/ml) + Epi (1000 μM) in complete RPMI, supplemented with 10% FBS and 2 mmol/L glutamine, at 37°C under a humidified atmosphere of 5% CO_2_ for 4h. After the cultivation period, the whole blood cells were labeled for surface markers and analysed with flow cytometry. Cell culture supernatants were collected and stored for IL-1β measurements.

### IL-1β Quantification by ELISA

Duplicate samples of the cell culture supernatants were analysed using Human IL-1β ELISA MAX Deluxe Set kit (Biolegend, UK) commercial assay, according to the manufacturer’s instructions. The samples were read at 450 nm on a microplate photometer HiPo MPP-96 (BioSan, Riga, Latvia). Results were calibrated with serial dilutions of known quantities of recombinant cytokines. The minimum detectable concentration for IL-1β was 0.5 pg/mL.

### Statistical Analyses

Data analysis was performed with GraphPad Prism 5.01 software (GraphPad Software, USA). All values are given as mean ± standard deviation (SD). Normal distribution was checked with Shapiro–Wilk’s W test. One-way ANOVA and Wilcoxon signed-rank tests as appropriate were used to estimate the effect of inducers within investigated groups. The Mann–Whitney test was used for the comparisons between studied groups. Principal component analysis (PCA) was performed using R software (version 4.0.5). Values of *P* < 0.05 were considered statistically significant.

## Results

### Spontaneous Expression of Surface Markers

**Figure 1:**
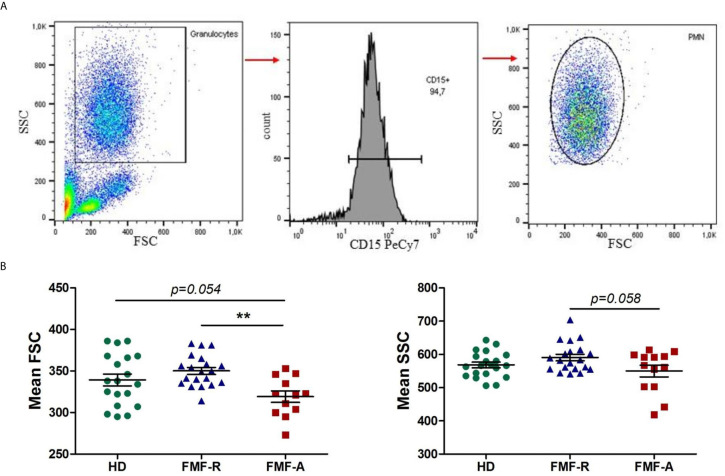
Analysis of morphological parameters of PMN. **(A)** Backgating strategy for PMN identification and FSC/SSC values. **(B)** Mean FSC and SSC parameters values of circulating PMNs in HD, FMF-R and FMF-A groups. **p < 0.01.

Peripheral blood PMNs were identified and described according to their FSC, SSC characteristics and CD15 expression. PMNs from the FMF-A group displayed low FSC and SSC parameters as measured by flow cytometry (**Figure 1**). Particularly, FMF-A PMNs showed lower size compared with both HD and FMF-R groups (*P* = 0.054 and *P* < 0.01, respectively), and tendency to the lower granularity compared with the cells from the FMF-R group (P=0.058). The cells from the FMF-A group also exhibited a higher percentage of SSC^hi^ cells compared to both HD and FMF-R (*P* < 0.05) (**Figures S1A, B**).

**Figure 2:**
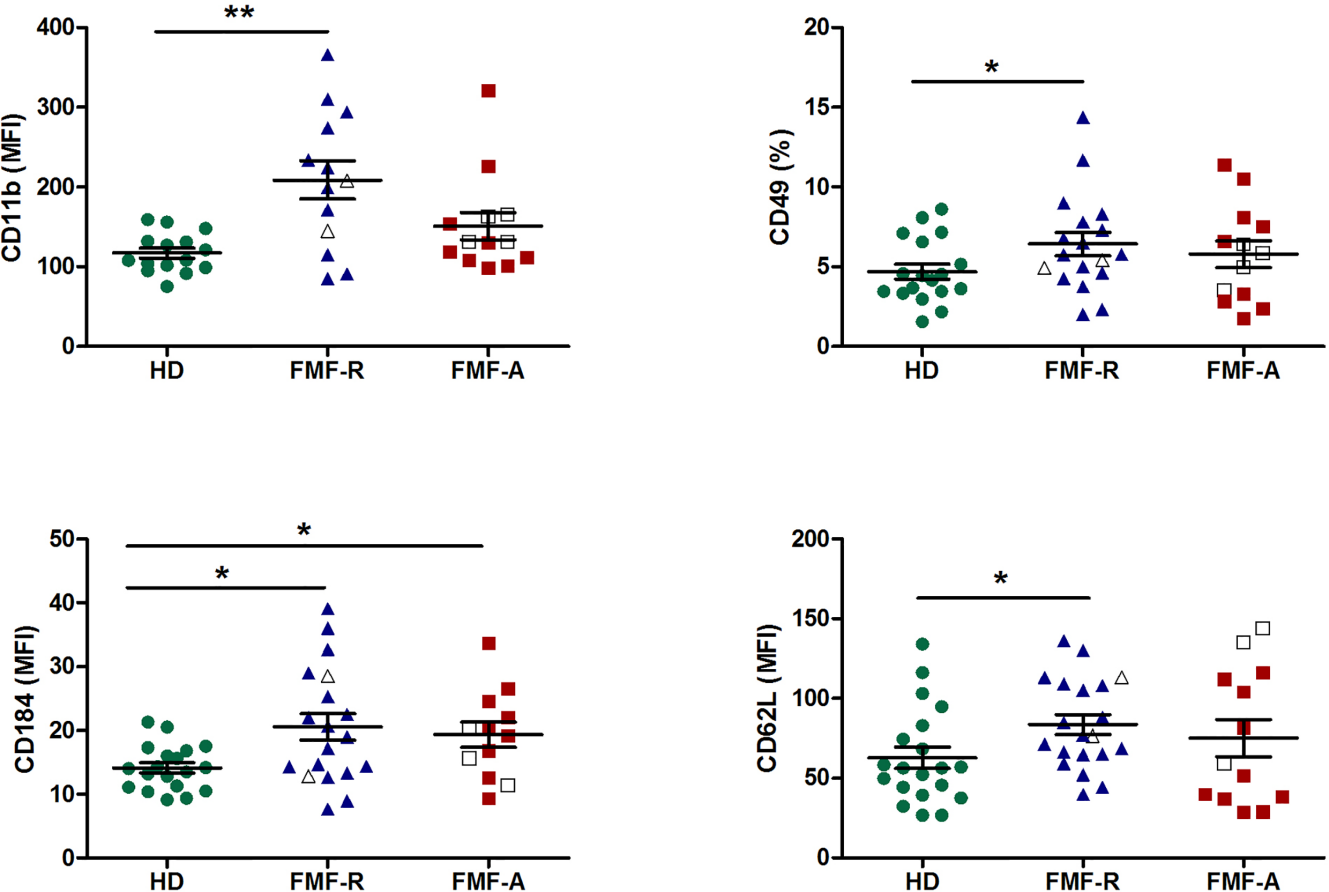
Surface expression of CD11b, CD49d, CD184 (CXCR4), and CD62L on circulating PMNs from HD, FMF-R and FMF-A patients. *p < 0.05; **p < 0.01.

Next, we examined the surface expression of CD11b, CD16, CD32, CD64, CD49d, CD62L, and CD184 (CXCR4) which is shown in **Figure 2**. Both FMF-R and FMF-A neutrophils displayed increased expression of CXCR4 (*P* < 0.05) compared to the HD group, while expression of CD11b (*P* < 0.01), CD62L (*P* < 0.05) and a percentage of integrin alpha subunit CD49-positive cells (*P* < 0.05) were increased only in the FMF-R group. A positive correlation between surface expression of CD11b and neutrophil percentage in patients from FMF-A group (*R* = 0.729, *P* = 0.01) was observed.

Subsets of circulating neutrophils with pro-inflammatory and anti-inflammatory features, were identified by the co-expression of CD11b and CD49d (**Figures S2A, B**), which are the markers able to characterize N1 (CD11b^lo^/CD49d^+^) and N2 (CD11b^hi^/CD49d^-^), respectively (21). There was an increase in CD11b^lo^/CD49d^+^ subset of PMNs with pro-inflammatory features in the FMF-R group compared to HD (*P* < 0.05) (**Figure S2C**).

**Figure 3:**
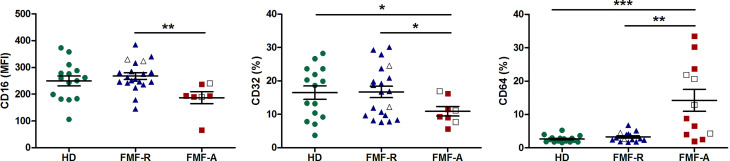
Surface expression of FcγRIII(CD16), FcγRII (CD32), FcγRI (CD64) on circulating PMNs from HD, FMF-R and FMF-A patients. *p < 0.05; **p < 0.01; ***p < 0.001.

Interestingly, while analysing expression of FcγRs on circulating PMNs, we observed multidirectional surface expression of these receptors. Particularly, a significant downregulation of low-affinity FcγRII (CD32) and FcγRIII(CD16) and upregulation of high-affinity FcγRI (CD64) expression in the cells from the FMF-A group were observed (**Figure 3**).

**Figure 4:**
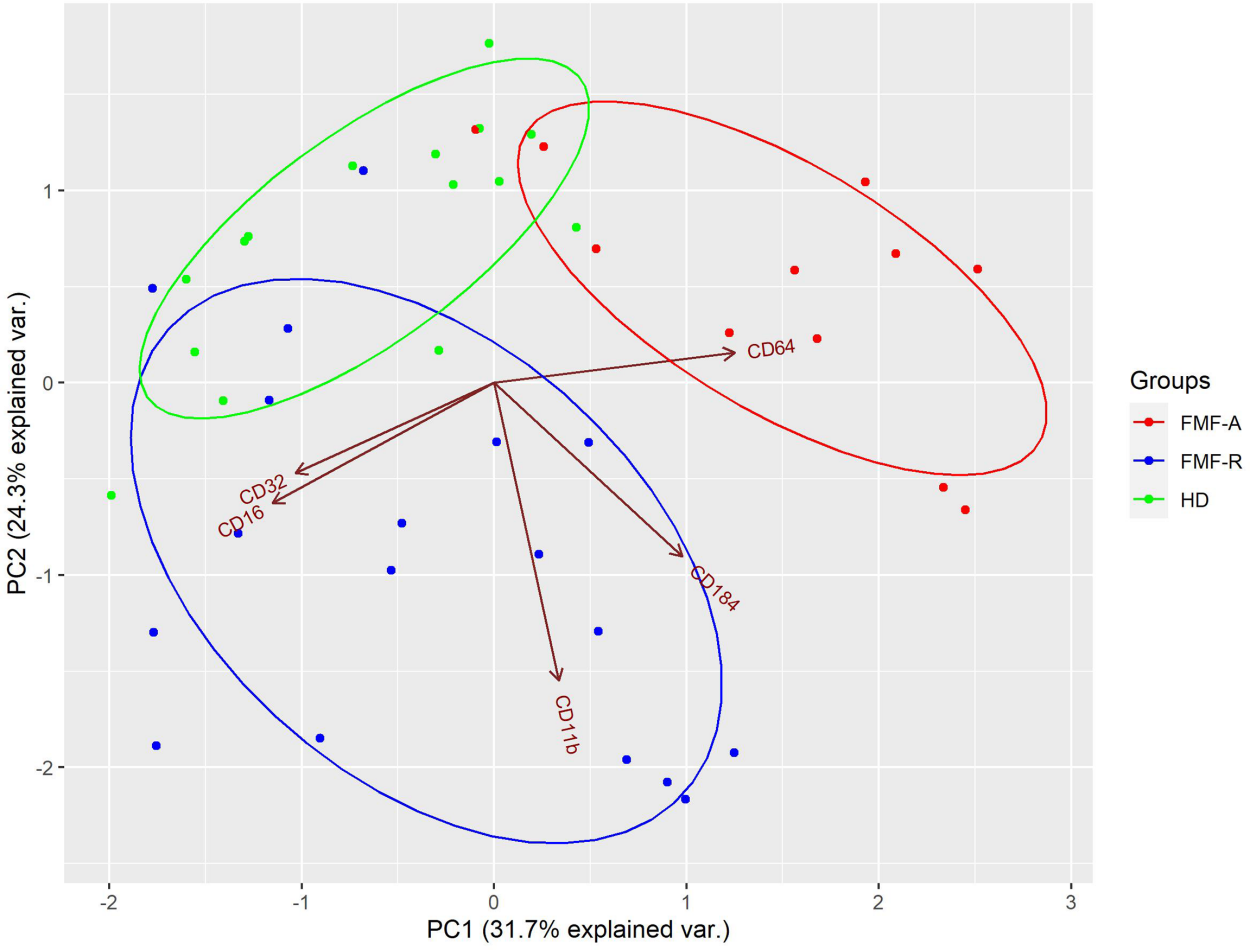
Principal component analysis (PCA) of PMN from HD (green), FMF-R (blue), and FMF-A (red), characterized by combination of CD11b, CD16, CD32, CD64, and CXCR4 expression. Each dot on bi-plot graph represents a single patient/control. The model loadings are represented by vectors and indicate how each surface marker contributes to the cell variability in a specific direction. Percentages represent variance captured by PC 1 and 2.

To determine if circulating neutrophils from all studied groups are distinct in expression profiles of surface markers and visualize the structure within the data, we used PCA. The analyses revealed clustering of three subpopulations (HD, FMF-R, FMF-A) with several dispersed cases, and few cases displayed in-between characteristics (2 HD, 3 FMF-R, and 2 FMF-A cases). In total, 56% of the cases were classified correctly. It is worth mentioning that overlapping of expression profiles was observed only between patients and controls not between FMF-R and FMF-A groups. Within studied markers, CD11b, CD16, CD32, CD64, and CXCR4 markers had the most consistent ability to correctly classify the cells from studied groups (**Figure 4**).

### Functional Activity of Circulating PMNs

Next, we addressed the question whether the patterns of FcRs expressed in the studied groups are associated with the phagocytosis. As appeared, phagocytic activity of PMNs was not different in both unstimulated and stimulated cells from all studied groups. Notably, PMNs from the FMF-R group failed to increase phagocytic activity in response to both fMLP and PGN which might be explained by the colchicine therapy received by the patients (**Figure S3A**).

As reactive oxygen species (ROS) can be found in higher amounts when neutrophils are activated, we used ROS as a parameter for neutrophil activity. Spontaneous oxidative burst of neutrophils in the FMF-A group was decreased compared to HD one (*P* < 0.05). A significant increase in ROS activity was detected upon fMLP stimulation in HD and FMF-R groups compared with unstimulated samples (*P* < 0.05 and *P* < 0.01, respectively). In the FMF-A group, fMLP-stimulated and unstimulated oxidative activity was reduced compared to the HD group (*P* < 0.05) and fMLP-stimulated compared to the FMF-R group (*P* < 0.05) (**Figure S3B**).

### Transmigration of Neutrophils From FMF Patients Activates the Cells

To determine whether mechanical forces induce increased activation of neutrophils, we mimicked conditions distinctive for the transendothelial migration. For this, we used 3µm pore Transwell inserts to allow neutrophils to migrate towards fMLP. In parallel, we cultured the cells in the absence or presence of fMLP in 24-well plates as a control (non-migrated) for the migrated cells. After 2h, we analysed the number of migrated cells, surface expression of CD11b, intracellular expression of IL-1β and production of IL-1β by migrated and non-migrated neutrophils. PMNs from FMF-A group displayed a tendency to a higher rate of transmigration compared to both HD and FMF-R cells (**Figure S4**).

**Figure 5:**
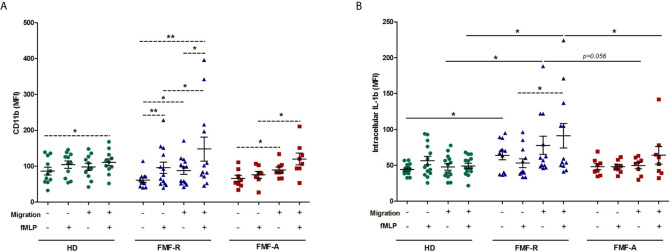
Activation patterns of migrated and non-migrated PMNs. PMNs were allowed to transmigrate through 3-μm pore Polyester Membrane Inserts or cultured in 24-well plates (non-migrated control cells) for 2h in absence or presence of fMLP. **(A)** Surface expression of CD11b (MFI), and **(B)** Intracellular expression of IL-1β (MFI), analysed by flow cytometry. *p < 0.05; **p < 0.01.

When analysing the effect of transmigration on cell activation, an increase in activation markers in the cells from the FMF-R group passing through the inserts was detected. When passing inserts, fMLP-induced cells from the FMF-R group revealed higher expression of IL-1β compared to those from the HD group (*P* < 0.05), except the non-migrated fMLP-stimulated cells. More notably, IL-1β expression within the FMF-R group was higher in migrated fMLP-stimulated PMNs compared to non-migrated ones (*P* < 0.05). When analysing surface CD11b expression, differences between studied groups were even more marked and exciting. Within the FMF-R and FMF-A groups, the migrated cells had a higher CD11b expression compared to the non-migrated cells (*P* < 0.05). FMF-R cells also responded to the fMLP stimulation more intensively as seen by up-regulated surface expression of CD11b on fMLP-stimulated cells compared to the unstimulated ones both after migration and without. Worth to mention that even without migration, cultured not-stimulated FMF-R cells exhibited up-regulation of IL-1β in comparison to those from the HD groups (*P* < 0.05) (**Figures 5A, B**).

Similarly to intracellular expression, the levels of released IL-1β were higher in the supernatants from the migrated fMLP-induced FMF-R cells compared with healthy ones (*P* < 0.05). However, the highest release of IL-1β was observed in the supernatants from the non-migrated FMF-R cells stimulated with fMLP which significantly higher compared to the FMF-A group (*P* < 0.05). Worth to mention that in the HD group, production of IL-1β by fMLP-stimulated PMNs in non-migrated was found to be higher than in migrated (**Figure S5**).

### Actin Dysfunctions in Neutrophils From FMF Patients

To analyse the kinetic of actin polymerization, we have measured the amounts of F-actin in neutrophils induced by fMLP at 5s, 15s, 30s, 60s, 120s, and 180s time points *in vitro*. PMNs from FMF-R group displayed the lowest actin polymerization activity at all-time points both in the absence or presence of fMLP, which might be explained by the received colchicine therapy by the patients. The cells from the FMF-A group stimulated with fMLP displayed a maximal F-actin polymerization earlier (at 5s) than in HD and FMF-R groups (at 30s) (*P* < 0.05 and *P* < 0.01, respectively). In contrast, plateau levels of polymerized F-actin content were reached earlier in HD and FMF-A neutrophils (after 1 min), while plateau levels in FMF-R were reached later (after 2 min) (**Figure S6**). The faster polymerization activity of the cells in acute flares is confirmed by a higher rate of transmigration observed in our study (albeit not significant).

### Production of IL-1β by Neutrophils From FMF-R Group Is Increased in a Time-Dependent Manner

**Figure 6:**
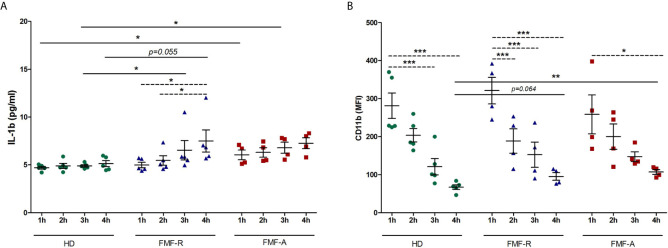
Analysis of time-dependent kinetics of PMNs activation. The cells were isolated from HD, FMF-R and FMF-A patients and cultured for 1h, 2h, 3h and 4h in induction-free media. After each cultivation period, PMNs and culture supernatants were analysed. **(A)** Production of IL-1β in supernatants of cultured PMNs as assessed by ELISA; **(B)** Surface expression of CD11b (MFI) analysed by flow cytometry. *p < 0.05; **p < 0.01; ***p < 0.001.

As was seen by migratory study, IL-1β expression in FMF-R cells was increased in induction-free media. To analyse time-dependent changes, we cultured PMNs continuously during 4h in RPMI media. The cells and supernatants were collected at 1, 2, 3, and 4h and analysed for IL-1β production and surface expression of CD11b. As shown in **Figure 6A**, cultivation of FMF-R and FMF-A cells has led to the increased production of IL-1β compared to the HD cells at different time points. Within the FMF-R group, an increase in IL-1β production was significant with the highest levels at 4h cultivation. In contrast, CD11b surface expression was gradually decreasing during the cultivation hours (**Figure 6B**). At 4h cultivation CD11b expression was higher on the cells from FMF-R and FMF-A groups compared to the HD group (*P* = 0.064 and *P* < 0.01, respectively).

### Epinephrine Is Not Significant Inducer of PMNs Activation in FMF

**Figure 7:**
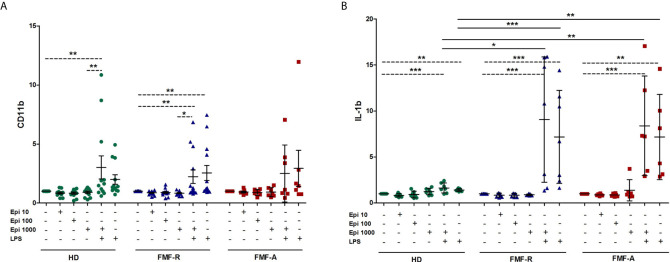
Effect of stress-related hormone Epinephrine (Epi) on PMNs activation. Whole blood cells were cultured in the absence or presence of Epi (10, 100, 1000 μM), LPS (100 ng/ml) or LPS (100 ng/ml) + Epi (1000 μM) for 4h. **(A)** Surface expression of CD11b (MFI) analysed by flow cytometry; **(B)** IL-1β production by whole blood cells was assessed in culture supernatants using ELISA. Data presented as fold change for stimulated CD11b and IL-1β production versus unstimulated ones. *p < 0.05; **p < 0.01; ***p < 0.001.

Since stress has been linked to the FMF flares, next we addressed the question whether stress hormone epinephrine (Epi) might be implicated in abnormal activation of FMF PMNs. For this, different concentrations of Epi were used to induce neutrophil’s response. As the analyses showed, there was no difference in expression of surface markers CD62L, CD11b on neutrophils exposed to different concentrations of epinephrine in all studied groups. In contrast, production of IL-1β was significantly increased in the supernatants from the cells exposed to LPS and LPS+Epi in FMF-R and FMF-A compared to those in the HD group (**Figure 7**). However, the differences in IL-1β concentrations between LPS-stimulated and LPS+Epi were not found which suggest that the observed effects of IL-1β were caused by LPS.

## Discussion

Despite pyrin function has been shown is an innate sensor for a number of pathogenic bacteria (5, 6, 8), it appeared that pyrin senses an event downstream of Rho modification, most likely involving the actin cytoskeleton modifications and do not directly detecting a microbial product (6, 7). Recently, Liston and Masters introduced a term named ‘homeostasis‐altering molecular processes’ (HAMPs), which describes the ability of innate immunity to recognize novel infections and trigger sterile inflammation (22). Pyrin inflammasome can sense cellular imbalance rather than a distinct pathogen enabling it to provide a defence to a large number of infections even novel (22, 23). The hypothesis that pyrin senses the changes in the cytoskeleton organization (6, 7, 23) is further confirmed by our study showing spontaneous and fMPL-induced activation of PMNs from FMF patients after transmigration as seen by the increased expression of CD11b and IL-1β. Transendothelial migration is a crucial step in the inflammatory response allowing PMNs exit circulation and enter a tissue. PMN migration is mediated by polarized shape changes and mechanical interactions with the extracellular tissues, driven by the cytoskeleton and associated proteins (24, 25). The selective activation of small Rho GTPases, including Rho, Rac, and Cdc42 results in the regulation of actin networks required to form varying structures involved in cell motility (26). Whether cytoskeletal activation caused by cell transmigration in the absence of virulent agents is able to assemble pyrin inflammasome or increased activation of FMF PMNs after transmigration was caused by another unidentified mechanism are unknown. The fact that colchicine blocks activation of the pyrin inflammasome after RhoA inactivation, favour that microtubule/actin dynamics might control or regulate pyrin activation (27).

In parallel, we analysed the population of circulating PMNs in terms of their immunophenotypes and functionality. As it turned out, circulating PMNs displayed heterogeneity as seen by the diverse phenotypes and FSC/SSC in the diseased groups. PMNs rapidly change their characteristics and behaviour as they get activated, aged, or primed by different mediators, and are able to polarize and produce alternative effector or immune‐regulatory molecules (28). In norm, neutrophils display different phenotypes based on the time they egress bone marrow and enter the circulation (fresh neutrophils) to the time they leave the circulation (aged neutrophils). Fresh neutrophils are characterized by CD62L^hi^CXCR4^lo^ phenotype, while aged neutrophils are CD62L^lo^CXCR4^hi^ (29, 30). CXCR4 is a chemokine receptor regulating the distribution and trafficking of neutrophils. Up-regulation of CXCR4 on aged PMNs was shown to promote their re-entering vasculature and migrating selectively back to the bone marrow in response to CXCL12 (31). Additionally, aged neutrophils are smaller, contain fewer granules and upregulate CD11b and CD49d, which promote their migration and adherence to inflamed tissues (29). In our study, FMF-A cells were characterized by the small cell size and granularity, increased number of the cells with SSC^hi^ parameters, and up-regulated CXCR4 receptor which reflects the presence of aged PMNs in the circulation of the patients in acute period. The aged PMNs with up-regulated CD11b and CXCR4 observed in the acute period of the disease may reflect their activated state and the direction of migration to the bone marrow resulting in the clearance of these leukocytes by resident macrophages (30). Here these aged neutrophils might be involved in the mobilization of fresh neutrophils from the bone marrow to replenish the number of circulating PMNs (32). FMF-R cells were of normal size and granularity and also characterized by the up-regulated CD11b, CD49d, CXCR4, and CD62L which suggests the mixed pool of the cells containing both fresh and aged fraction of the cells in the circulation. Heterogeneous population of FMF-R cells might also reflect a high turnover of circulating PMNs and their activated status even in the absence of acute flares. Results of the current study may explain why tissue-infiltrated PMNs do not cause immune-mediated tissue damage. Rather, sterile tissue-infiltrated aged PMNs undergo reverse transmigration migrating back into the circulation and then to the bone marrow *via* CXCR4 (29, 33, 34). This phenomenon is a form of biologic recycling, an event that is unlikely to occur in infections (34).

In addition to the changes in the heterogeneity of neutrophils, a down-regulation of CD16 (FcγRIII) and CD32 (FcγRIIA) in PMNs from FMF-A group were observed, which also confirms aged/activation status of the cells during acute flares. Modulation of FcγRII- and FcγRIII-induced cell activation might be achieved by receptor internalization or shedding of the extracellular portion of the FcγRs. The cleavage of FcRs from the cell surface has been shown after neutrophil stimulation and during neutrophil apoptosis (35). It was shown that cross-linking of FcγRIIA (spontaneously expressed on neutrophils) leads to the activation, degranulation, and production of inflammatory mediators and ROS (35). A decrease in expression of FcγRIII was shown in heat-stressed neutrophils which was suggested to contribute to the anti-inflammatory signalling at inflamed sites and preceded the development of spontaneous apoptosis (36). In contrast to FcγRII and FcγRIII, FcγRI was significantly high in the FMF-A group which has been shown to be strongly upregulated in the presence of inflammatory cytokines and reflects disease activity in numerous inflammatory conditions (37, 38). The reason for multidirectional expression FcγRs is unknown. A possible explanation might reside in differential differences in monomeric/multimeric IgGs or other factors in the blood of FMF patients during acute flares, however experimental data to support this hypothesis are missing.

In conclusion, the current study raised the possibility of heightened sensitivity of mutated pyrin inflammasome towards cytoskeletal modifications in the absence of pathogens. Whether pyrin in turn modulates biomechanics of the cells is unknown. Further studies should be directed towards understanding whether cytoskeleton perturbations alone are able to influence pyrin phosphorylation state.

## Data Availability Statement

The original contributions presented in the study are included in the article/**Supplementary Material**. Further inquiries can be directed to the corresponding author.

## Ethics Statement

The studies involving human participants were reviewed and approved by Ethical Committee of the Institute of Molecular Biology NAS RA (IRB IORG0003427). Written informed consent to participate in this study was provided by the participants’ legal guardian/next of kin.

## Author Contributions

GM conceived the study and planned the experiments, interpreted the data, and wrote the manuscript. NM and GA collected the patient samples and clinical data. AM, DP, and SG performed the analysis. AM and DP performed the statistical, data mining analysis and designed the figures. AM, DP, and GA revised the manuscript. All authors contributed to the article and approved the submitted version.

## Funding

This work was supported by a State Committee Science MES RA, in frame of the research project no. SCS 20RF-112.

## Conflict of Interest

The authors declare that the research was conducted in the absence of any commercial or financial relationships that could be construed as a potential conflict of interest.
