# Supplementary material for: Transmigration of Neutrophils From Patients With Familial Mediterranean Fever Causes Increased Cell Activation
Source: Front Immunol. 2021 May 17;12:672728. doi: 10.3389/fimmu.2021.672728 (PMC8165278; doi:10.3389/fimmu.2021.672728)

**Supplementary data**

**Transmigration of neutrophils from patients with familial Mediterranean fever causes increased cell activation**

**Fig S1.** **High SSC neutrophils in the whole blood of FMF patients and healthy subjects. A)** Gating strategy of SSC^hi^ neutrophils (representative zebra-plot). The percentage of SSC^hi^ PMNs were taken from CD15+ cells; **B)** The percentages of SSC^hi^ cells in the HD, FMF-R, and FMF-A groups. **p* < 0.05; Mann–Whitney test.


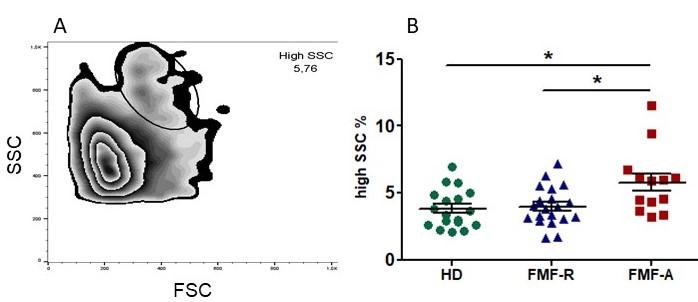


**Fig S2. Gating strategy of N1 and N2 PMNs. A)** and **B)** representative dot-plots with prevalence of N1 (CD11b^lo^/CD49d^+^) and N2 (CD11b^hi^/CD49d^-^) populations, respectively; **C)** The percentages of N1 and N2 cells in the HD, FMF-R, and FMF-A groups. **p* < 0.05.


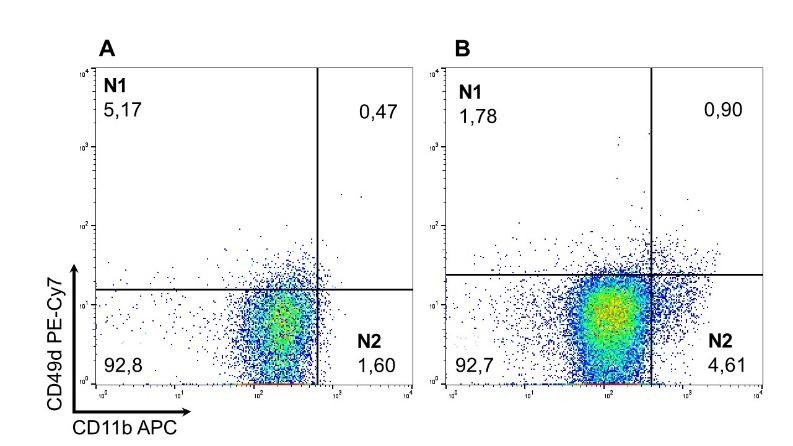

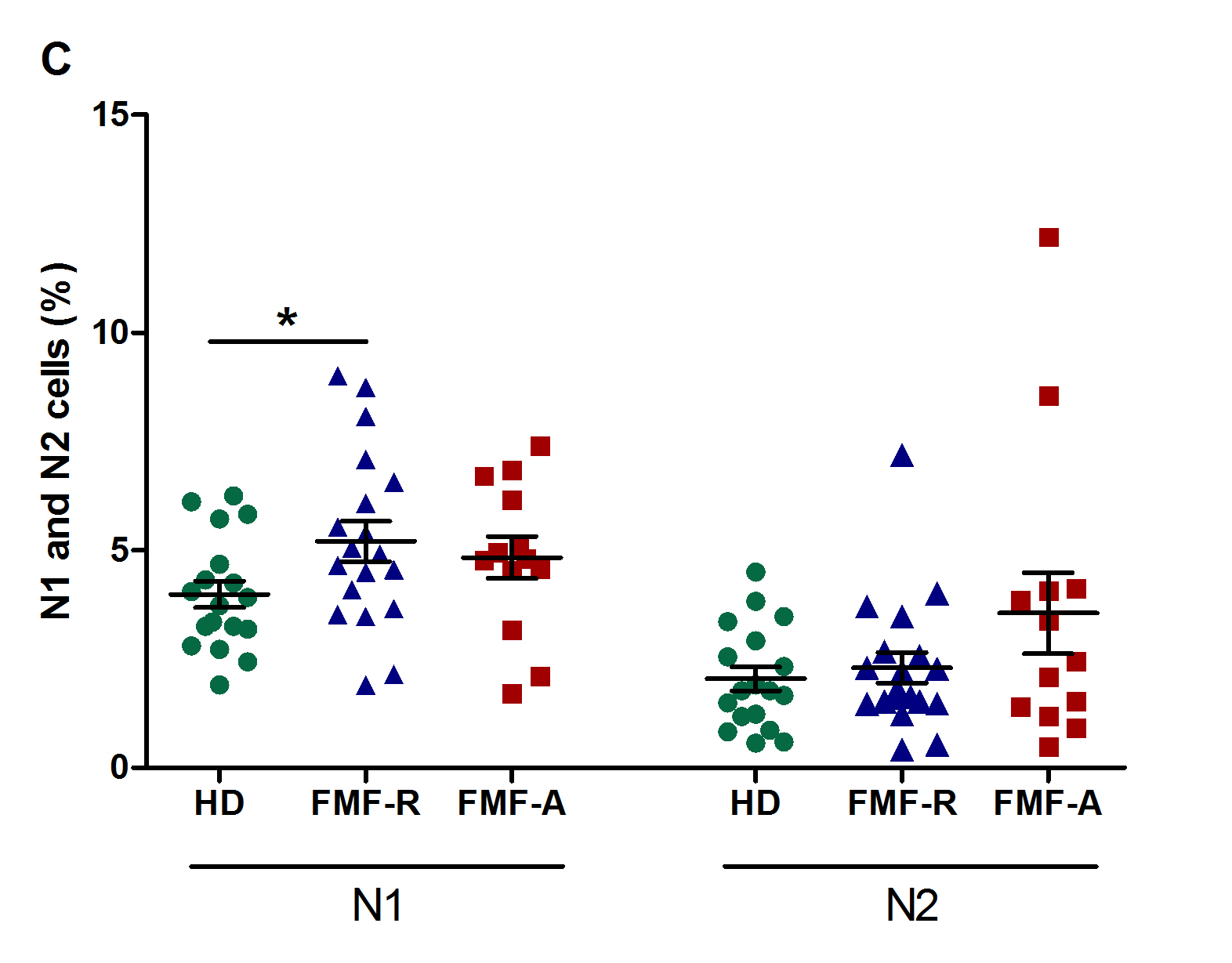


**Figure S3.** Functional activity of circulating PMNs from HD, FMF-R and FMF-A patients. **A)** Phagocytic activity was determined as the percentage of phagocytizing PMNs, following pre-stimulation with LPS or PGN. **B)** ROS generation in fMLP-stimulated and unstimulated PMNs presented as mean fluorescence intensity (MFI); **p* < 0.05; ***p* < 0.01; ****p* < 0.001.


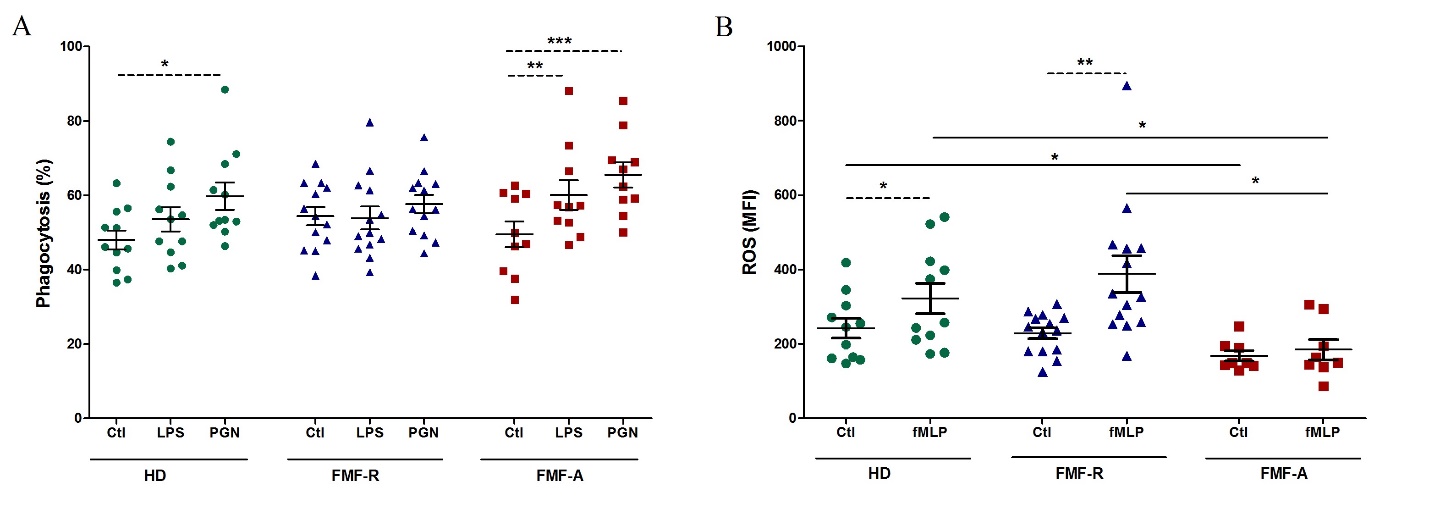


**Figure S4.** Transmigration rate of PMNs through 3-μm pore Polyester Membrane Inserts under static conditions. Migration rate was counted after 2 hours and expressed as ratio of the cells migrated in the presence of fMLP to the cells migrated in the absence of fMLP for each studied group.


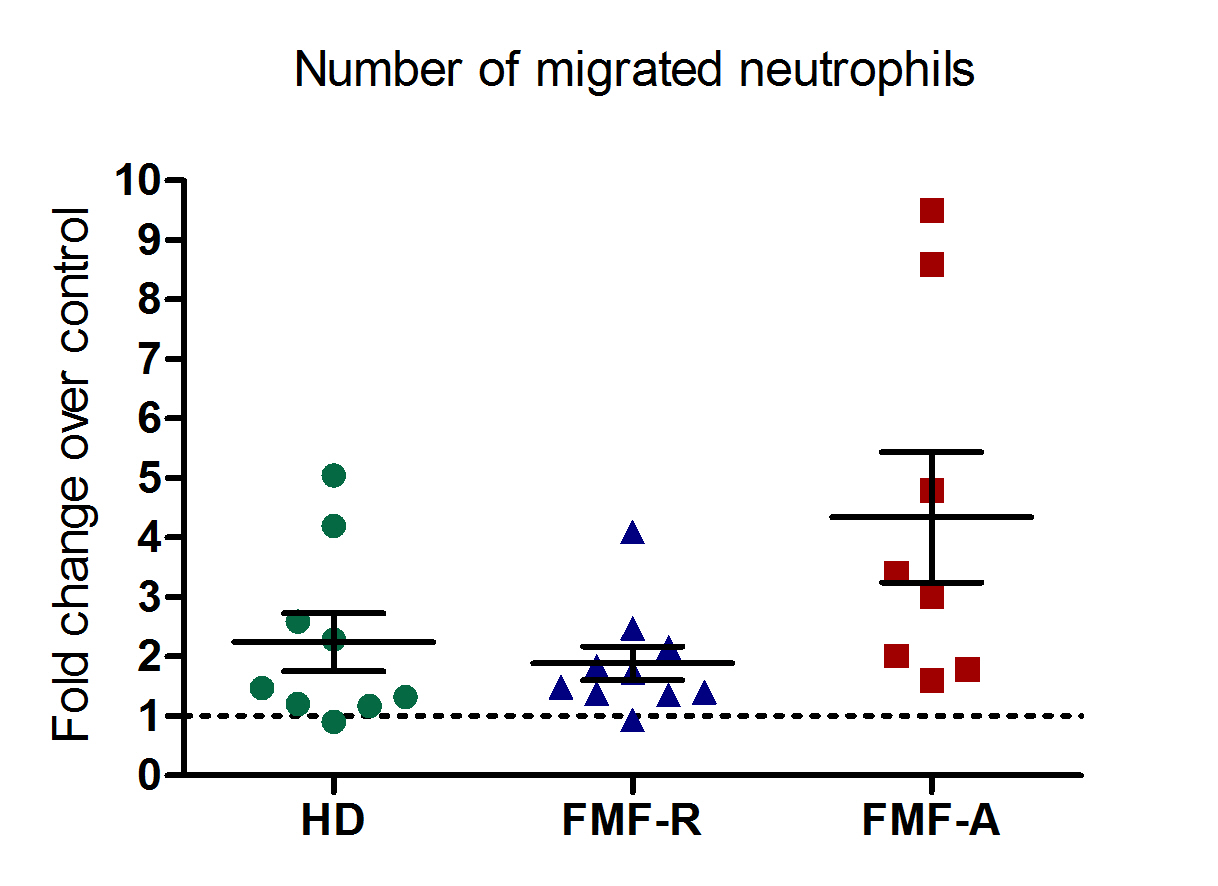


**Figure S5.** Production of IL-1β in the supernatants collected from the lower compartment of Transwell chamber after PMNs migration and the well with cultured (non-migrated) PMNs in the absence or presence of fMLP and analyzed with ELISA. IL-1β levels are reported as pg/ml. **p* < 0.05.


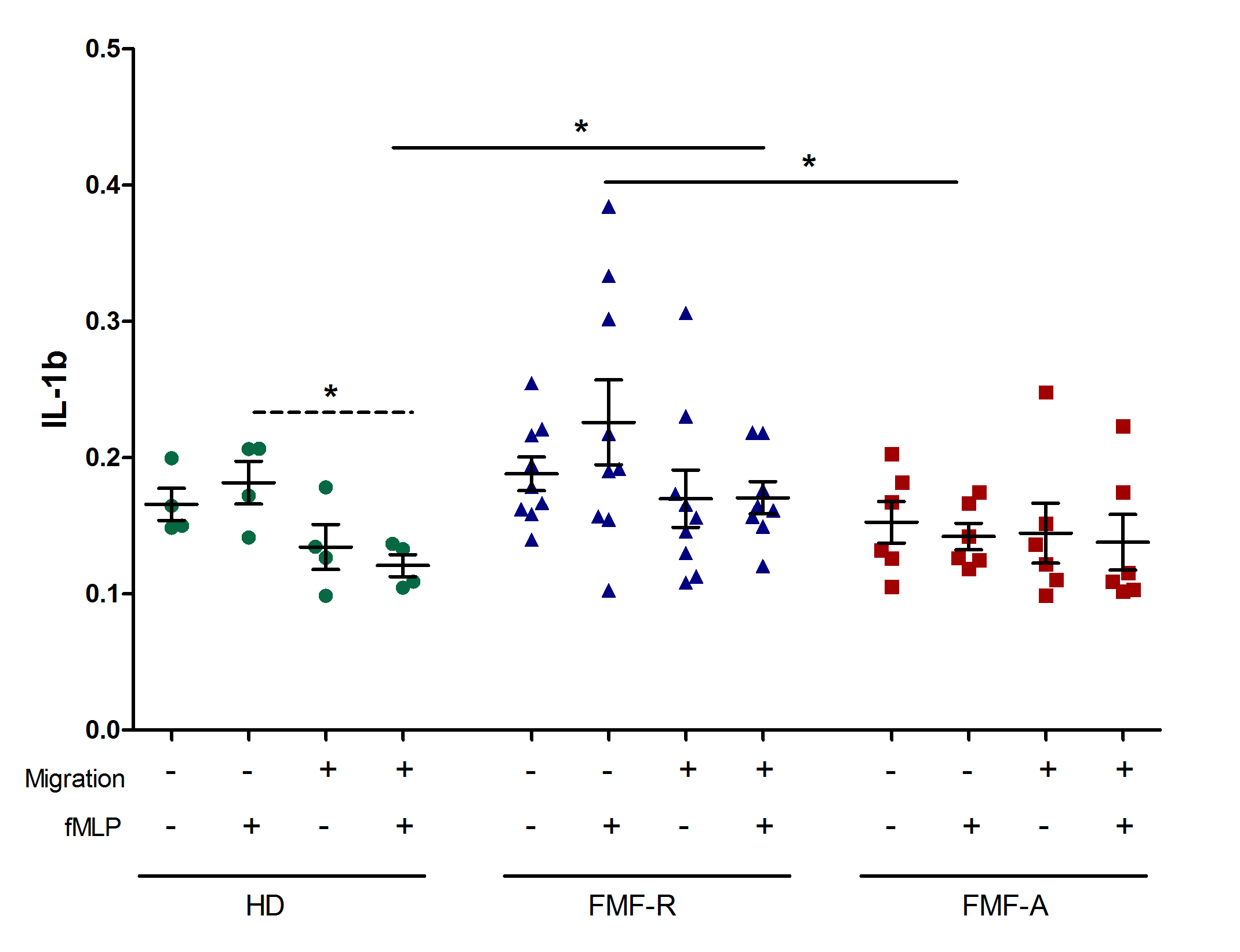


**Figure S6.** F-actin polymerization activity of isolated PMNs from the blood of HD, FMF-R and FMF-A patients, measured at basal level (0s) and in the absence (dotted line) or presence of fMLP (continuous line) for 5s, 15s, 30s, 60s, 120s, and 180s. Polymerization activity is presented as MFI of Phalloidin. ***p* < 0.01 FMF-A *vs* FMF-R, #*p* < 0.05 FMF-A vs HD.


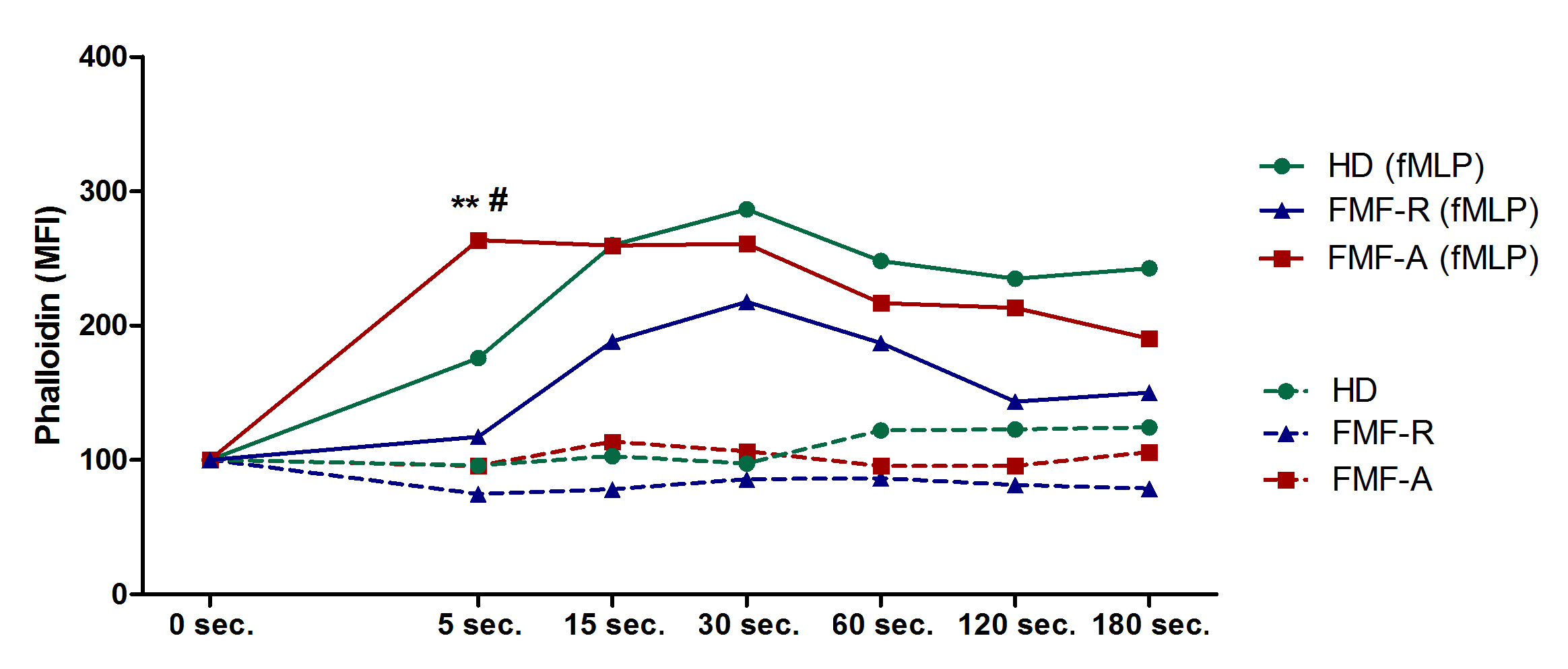

Supplement: Supplementary file 1 [file DataSheet_1.docx]
